# Comparative Analysis of Maintenance Treatments in Patients with Newly Diagnosed Advanced Ovarian Cancer After First-Line Platinum-Based Regimens

**DOI:** 10.3390/cancers17223714

**Published:** 2025-11-20

**Authors:** Lorenzo Gasperoni, Luca Cancanelli, Andrea Ossato, Luna Del Bono, Stefano Vecchia, Caterina Fontanella, Vera Damuzzo, Andrea Messori

**Affiliations:** 1 Oncological Pharmacy Unit, IRCCS Istituto Romagnolo per lo Studio dei Tumori (IRST) “Dino Amadori”, 47014 Meldola, Emilia Romagna, Italy; 2 Hospital Pharmacy Department, Azienda Ulss 2 Marca Trevigiana, 31100 Treviso, Italy; 3 Italian Society of Clinical Pharmacy and Therapeutics (SIFaCT), 10123 Turin, Italy; 4 UOC Territorial Pharmaceutical Service, Azienda ULSS 8 Berica, 36100 Vicenza, Italy; 5 Department of Pharmacy, School of Specialization in Hospital Pharmacy, University of Pisa, 56126 Pisa, Italy; 6 Hospital Pharmacy Unit, Ospedale Guglielmo da Saliceto, 29121 Piacenza, Italy; 7 Oncology and Oncohematology Unit, Vittorio Veneto Hospital, AULSS2 Marca Trevigiana, 31029 Vittorio Veneto, Italy; 8 HTA Unit, Regional Health Service, 50139 Florence, Italy; 9 Italian Society of Clinical and Therapeutic Pharmacy, 10123 Turin, Italy

**Keywords:** advanced ovarian cancer, PARP-inhibitors, IPDfromKM, indirect comparison, meta-analysis

## Abstract

Background: Poly (ADP-ribose) polymerase inhibitors (PARPi) are the standard of care for first-line maintenance in advanced ovarian cancer, but their benefit varies by BRCA and homologous recombination deficiency (HRD) status, and no head-to-head comparisons are available. Methods: We conducted an indirect comparison of PARPi regimens using reconstructed individual patient data (IPD) from Kaplan–Meier curves of phase III randomized trials (SOLO1, PRIMA, PAOLA1, ATHENA, FLAMES). Progression-free survival (PFS) was the primary endpoint; overall survival (OS) was exploratory. Subgroups were defined as BRCA−mutated (BRCA+), BRCA−/HRD+, and BRCA−/HRD−. Safety outcomes were assessed through a network meta-analysis of adverse drug reactions (ADRs). Results: In BRCA+ patients, olaparib + bevacizumab achieved the largest PFS improvement (HR = 0.27; 95%CI: 0.19–0.39), followed by olaparib monotherapy, while niraparib performed significantly worse. In BRCA−/HRD+, olaparib + bevacizumab was superior to niraparib and rucaparib, with restricted mean survival time (RMST) gains of 3–4 months. In BRCA−/HRD−, PARPi produced only a modest benefit, with no advantage over bevacizumab monotherapy. Exploratory OS analysis confirmed long-term survival with olaparib in BRCA+ but not in the other subgroups. Safety analysis indicated olaparib had the most favorable hematological profile, while niraparib was associated with the highest rates of severe anemia, thrombocytopenia, and neutropenia, despite showing lower gastrointestinal toxicity and fatigue incidence. Conclusions: PARPi efficacy depends strongly on BRCA and HRD status. Olaparib-based regimens provide the greatest clinical benefit with acceptable safety in BRCA+ and HRD+ disease, whereas PARPi appear to be of limited value in HRD-negative ovarian cancer.

## Simple Summary

Poly (ADP-ribose) polymerase inhibitors (PARPi) are standard first-line maintenance therapy in advanced ovarian cancer, but their benefit is strongly influenced by BRCA status and homologous recombination deficiency (HRD), and no direct head-to-head trials exist. We performed an indirect comparison using reconstructed individual patient data from Kaplan–Meier curves of phase III studies (SOLO1, PRIMA, PAOLA1, ATHENA, FLAMES). The primary endpoint was progression-free survival (PFS), while overall survival (OS) was exploratory. Subgroups were BRCA−mutated (BRCA+), BRCA−/HRD+, and BRCA−/HRD−. Safety was assessed through a network meta-analysis of adverse events. In BRCA+ patients, the largest PFS benefit was observed with olaparib plus bevacizumab, followed by olaparib monotherapy, while niraparib performed worse. In BRCA−/HRD+ disease, olaparib plus bevacizumab outperformed niraparib and rucaparib, with restricted mean survival time gains of 3–4 months. In BRCA−/HRD− patients, PARPi yielded only modest benefits, showing no advantage over bevacizumab alone. Exploratory OS analysis confirmed durable survival with olaparib in BRCA+ but not in other subgroups. Regarding safety, olaparib demonstrated the most favorable hematologic profile, whereas niraparib was linked to higher rates of severe anemia, thrombocytopenia, and neutropenia, although it showed lower gastrointestinal toxicity and fatigue. In conclusion, PARPi efficacy is highly dependent on BRCA and HRD status: olaparib-based regimens provide the greatest clinical advantage with manageable safety in BRCA+ and HRD+ disease, while their value in HRD-negative ovarian cancer remains limited.

## 1. Introduction

Ovarian cancer is the fourteenth leading cause of cancer death worldwide, accounting for over 200,000 deaths per year, and the fifth leading cause of cancer death among women in the United States [1,2]. The treatment approach for newly diagnosed advanced ovarian cancer consists of cytoreductive surgery and systemic therapy [3,4], and residual tumor at the end of surgery represents one of the most important prognostic factors [1]. However, half of all patients present with distant metastases at diagnosis, and most patients treated with curative intent experience relapse within three years [5]. The need for first-line treatments that maintain a response to platinum therapy over time has encouraged the development of effective maintenance therapies that are potentially capable of delaying relapse. Adding bevacizumab to platinum therapy, followed by bevacizumab alone, led to prolonged progression-free survival (PFS) but no improvement in overall survival (OS) [6,7,8,9]. Approximately half of high-grade serous ovarian carcinomas are characterized by pronounced genomic instability, attributable to alterations in genes governing homologous recombination repair (HRR). In the presence of BReast CAncer genes (BRCA) mutations or defects within the homologous recombination repair (HRR) pathway (Homologous Recombination Deficiency—HRD), tumor cells become dependent on error-prone DNA repair processes resulting in heightened genomic instability, which, paradoxically, increases tumor susceptibility to DNA-damaging agents (e.g., platinum compounds) and to PARPi (PARP-i) through the mechanism of synthetic lethality [10]. The introduction of PARPI as a therapeutic option has expanded the treatment landscape for ovarian cancer [11], and PARPi currently represent the standard of care for first-line maintenance treatment [12,13,14,15,16,17,18,19,20]. In addition, the introduction of PARPi has highlighted differences in efficacy among subpopulations, confirming the expression of BRCA and HRD genes as important stratification biomarkers for optimal clinical decision. The current treatment scenario reimbursed by the Italian National Health Service envisages the potential use of olaparib as monotherapy only for patients with a BRCA mutation, and olaparib in combination with bevacizumab for patients with no BRCA mutation and HRD. Niraparib and rucaparib could potentially be used to treat patients regardless of their mutational status. However, as there is no direct comparison between the various PARPi in different subpopulations, several questions remain unanswered. For example, is the use of PARi such as niraparib and rucaparib in HR-proficient patients clinically appropriate? Are currently available PARPi equally effective? Could the combination of bevacizumab and olaparib be more beneficial for patients with BRCA mutations than monotherapy with other PARPi? In a scenario with many new features, such as the one described, it is crucial to select the most effective molecularly targeted therapy to maintain disease control as long as possible. This study aims to indirectly compare currently available treatments and combinations for first-line maintenance treatment of ovarian cancer. Some systematic reviews and meta-analyses on this topic have recently been published in the literature [21,22,23]. However, this is the first study to compare PARPi using individual patient data (IPD) reconstruction, which also allows for long-term survivors to be accounted for. To this end, we used the IPDfromKM methodology to reconstruct IPD from the main randomized clinical trials on maintenance therapy with PARPi and provide an indirect comparison of the differential efficacy of the various treatment options in BRCA+, HRD+/BRCA− and BRCA−/HRD− subgroups. In addition to this methodological novelty, our analysis was based on the latest results from the main randomized controlled trial (RCT), including the recently published FLAMES RCT. We also explored safety and overall survival comparisons [24].

## 2. Materials and Methods

### 2.1. Literature Search

We conducted a systematic search of the PubMed database to identify RCTs that were relevant to our analysis. The last search was performed on 2 July 2025. The search strategy was as follows: [(“Ovarian Cancer” OR “ovarian carcinoma”) AND (“Parp inhibitor” OR “PARPi” OR “parpi” OR Olaparib OR Niraparib OR Rucaparib OR Veliparib OR Senaparib OR Talazoparib OR Pamiparib) AND (“first-line” OR “first-line” OR maintenance OR “newly diagnosed”)]. The selection process adhered to the PRISMA guidelines [25]. The inclusion criteria were as follows: (a) phase III RCT phase III; (b) first-line maintenance treatment of advanced ovarian cancer; (c) availability of PFS outcomes; and (d) data presented through Kaplan–Meier (KM) survival curves. We excluded studies in which KM curves reported survival data starting not from the beginning of the maintenance phase but also including the previous chemotherapy treatment (VELIA and FIRST RCTs [26,27]). To avoid duplication of data, the most recent and complete version was selected for inclusion when multiple publications from the same trial were available.

### 2.2. Reconstruction of Individual Patient Data

The IPDfromKM method [24] was used to reconstruct IPD from the KM survival curves of the treatment and control arms reported in the selected RCTs. The KM curves were digitized using WebPlotDigitizer (version 4.7; available online at https://apps.automeris.io/wpd/, accessed 18 August 2025. Distance: 20, Δx and Δy = 15). The extracted X–Y coordinates, together with the number of enrolled patients and events, were then processed using the IPDfromKM package (version 1.2.3.0; last updated 22 March 2022, available at https://www.trialdesign.org/one-page-shell.html#IPDfromKM (accessed on 15 Ocober 2025)). This generated a reconstructed dataset providing survival times (defined as the interval from trial enrollment to the last available follow-up) and patient outcomes, which were categorized as alive/censored or dead/progressed. This procedure yielded reconstructed IPD for each RCT arm. The entire procedure (digitalization + IPD reconstruction) was performed in duplicate by two independent researchers and results are compared for consistency to minimize the risk of manual errors.

### 2.3. Study Design and Data Analysis

This analysis aimed to evaluate the relative efficacy of maintenance treatments for patients with newly diagnosed advanced ovarian cancer following first-line platinum-based chemotherapy, with PFS as the primary endpoint and OS assessed as an exploratory outcome. The regimens evaluated included the PARPi olaparib, niraparib, rucaparib, and senaparib, as well as a treatment arm combining olaparib with bevacizumab.

KM curves for PFS were reconstructed for each trial arm, while OS curves were available only for SOLO1, PAOLA1, and PRIMA trials. Subgroup analyses were conducted according to molecular characteristics of the primary tumor (BRCA+, BRCA−/HRD+, BRCA−/HRD−). For comparative analyses, placebo was adopted as the common comparator across the included trials. All patients receiving placebo across the included studies were pooled and analyzed together to constitute the control group. As in the PAOLA 1 study, the control group consisted in an active treatment (bevacizumab), we assessed in each subgroup of patients whether the use of bevacizumab induced the control group to behave differently from placebo-control arms. To assess this possible inconsistency of control groups across the included studies, the likelihood ratio test and the concordance statistic were employed, following established methods for evaluating inter-study heterogeneity. If heterogeneity was present, as in the HRD−/BRCA− population, the bevacizumab arm was considered separately. Treatment effects were estimated using a Cox proportional hazards model, with the results expressed as hazard ratios (HR) and 95% confidence intervals (CI). Homogeneity of the control groups across the trials was evaluated using likelihood ratio tests and concordance statistics. Indirect pairwise comparisons among active regimens were also performed through Cox regression models. Restricted Mean Survival Time (RMST) was calculated as an additional measure of treatment effect for PFS only, with KM curves truncated at 33, 30, and 26 months for the BRCA+, BRCA−/HRD+, and BRCA−/HRD− subgroups, respectively, which corresponds to the minimum follow-up time available across the included RCTs. As PFS results at longer follow-up time were available for SOLO1 and PRIMA, for these studies a further comparative analysis based on RMST at 75 months was performed. All statistical analyses were performed with R software (version 4.3.2).

Finally, the most frequently reported adverse drug reactions (ADRs), both of any-grade and grade ≥ 3, were collected from the included trials. A frequentist network meta-analysis with a random-effects model was conducted using MetaInsight software [28] to estimate the relative risk (RR) of developing each ADR, with placebo defined as the reference comparator. In addition, forest plots were generated using the Meta-Mar software (version 4.0.2) [29].

## 3. Results

### 3.1. Indirect Comparison Efficacy Analysis

**Figure 1:**
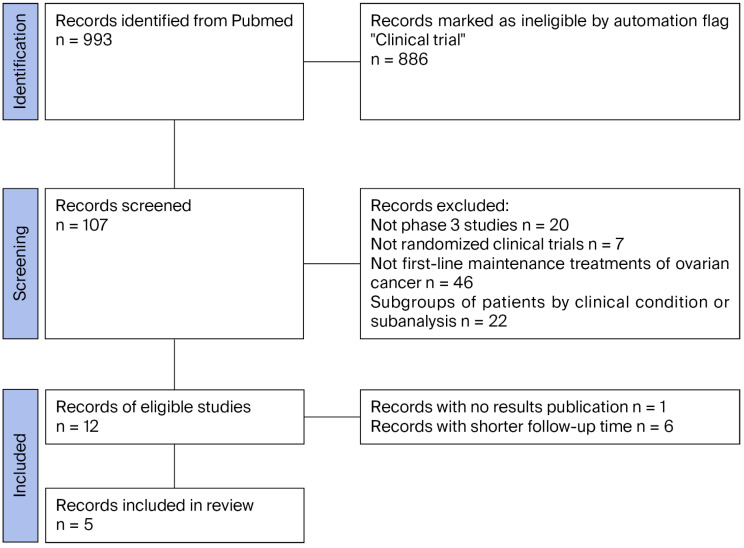
PRISMA flowchart of the process of trial selection.

A comprehensive literature search retrieved 993 records, which were screened based on predefined inclusion and exclusion criteria to capture the most recent RCTs evaluating maintenance therapy with PARPi in patients with newly diagnosed advanced ovarian cancer following first-line platinum-based chemotherapy. The selection process, illustrated in Figure 1 according to PRISMA recommendations, resulted in the identification of five RCTs eligible for indirect comparison analysis on progression-free survival (PFS) outcomes (Table 1). Results from the VELIA and FIRST studies have been excluded as these studies reported PFS starting from the first cycle of chemotherapy rather than from the first cycle of maintenance therapy with a PARP inhibitor. This would have resulted in an artificial elongation of PFS times compared to other RCTs.

The trials included in the analysis enrolled patients with newly diagnosed ovarian cancer, mainly FIGO stage III or IV, who underwent primary or interval cytoreductive surgery and received either neoadjuvant or adjuvant chemotherapy with platinum-based regimens. The populations included in the studies are homogeneous in terms of histology, stage, and visible residual disease. The FLAMES study included only Asiatic patients, while the proportion of this ethnicity in the other RCTs varies, being 19% in the ATHENA trial or otherwise not disclosed.

The SOLO1 RCT compared olaparib maintenance therapy in patients with a BRCA mutation. The other RCTs included a more heterogeneous population composed of patients with BRCA mutations, patients with HR deficiency, and patients without BRCA or HR alterations (wild-type, WT).

The ATHENA, PRIMA, and FLAMES RCTs investigated the efficacy of maintenance therapy with the PARPi niraparib, rucaparib, and senaparib, respectively. All studies compared the efficacy of PARPi to a placebo, which was used as the common comparator in our analysis, except for in the PAOLA1 and ATHENA study; all patients enrolled in PAOLA1 RCT and 20% of patients included in the ATHENA trial also received bevacizumab alongside either with PARP inhibitor or placebo during the maintenance phase.

As there were three main subgroups present in the cohorts that could be classified based on BRCA and HR status, we decided to split our analysis into three parts, considering patients with BRCA mutations (BRCA+), patients without BRCA mutation but HR deficient (BRCA−/HRD+), and patients wild-type for BRCA and HR proficient (BRCA−/HRD−). BRCA+ populations were present in all studies, while results on BRCA−/HRD+ were derived from ATHENA, PAOLA1, and PRIMA RCTs. Data for BRCA−/HRD− were present only for ATHENA and PRIMA studies.

**Table 1:** Summary of the main clinical characteristics of patients treated with PARPi included in the analysis. Pts: patients; HRD: homologous recombination deficient; CR: complete response; PR: partial response; CT: chemotherapy; NA: not available. Median PFS and median OS are expressed in months.

Trial [References]	Treatments Under Comparison	N. of Pts	FIGO III/IV	% CR/PR to CT	% PDS/IDS	% BRCA+	% HRD+	Median PFS	HR for PFS (95% CI)	Median OS	HR for OS (95% CI)
SOLO1 [12,13,14]	Olaparib vs. Placebo	260 131	85/15% 80/20%	82/18% 82/18%	62/36% 65/33%	100% 100%	100% 100%	56 13.8	0.33 (0.25–0.43)	NR 75.2	0.55 (0.40–0.76)
PAOLA1 [15,16]	Olaparib + Bevacizumab vs. Placebo + Bevacizumab	537 269	70/30% 69/31%	20/26% 20/28%	50/42% 51/41%	30% 30%	47% 49%	22.1 16.6	0.59 (0.49–0.72)	56.5 51.6	0.92 (0.76–1.12)
ATHENA [17]	Rucaparib vs. Placebo	427 111	76/24% 70/30%	17/18% 10/20%	49/51% 49/51%	21% 22%	43% 44%	20.2 9.2	0.47 (0.31–0.72)	NR 46.2	0.83 (0.58–1.17)
PRIMA [18,20]	Niraparib vs. Placebo	487 246	65/35% 64/36%	70/30% 70/30%	32/65% 32/67%	32% 56%	34% 60%	13.8 8.2	0.62 (0.50–0.76)	46.6 48.8	1.01 (0.84–1.23)
FLAMES [11]	Senaparib vs. Placebo	271 133	69/31% 74/26%	87/13% 89/11%	NA	35% 33%	75% 66%	NR 13.6	0.43 (0.32–0.58)	NR NR	NA

Abbreviations: NR, not reported.

Although each group was homogeneous in terms of genetic profile, there were some differences due to the different proportions of complete responses achieved after chemotherapy and the addition of bevacizumab to PARPi in all patients from the PAOLA1 study and a certain proportion of patients from the ATHENA RCTs. Therefore, an analysis of heterogeneity was conducted to ascertain whether the control groups in the RCTs behaved similarly in terms of PFS. The heterogeneity test revealed no substantial heterogeneity between the PFS of the control arms in the BRCA+ cohort (likelihood ratio test = 7.03 with four degrees of freedom, *p* = 0.1) and in the BRCA−/HRD+ population (likelihood ratio test = 3.14 with two degrees of freedom, *p* = 0.2). Conversely, in the BRCA−/HRD− population, the use of bevacizumab in the control arms of the PAOLA1 RCT increased the heterogeneity of the analysis. Therefore, in this subgroup, we opted to use the pooled reconstructed IPD from the ATHENA and PRIMA RCTs as the control arms and to consider the bevacizumab arm of the PAOLA1 study as an independent treatment arm.

PFS Kaplan–Meier curves of control arms of the three groups are reported in Supplementary Figure S1.

After ensuring the comparability of the RCTs, we indirectly compared the efficacy of maintenance treatment with PARPi in BRCA+, BRCA−/HRD+, and BRCA−/HRD− subgroups. The primary endpoint was PFS.

**Figure 2:**
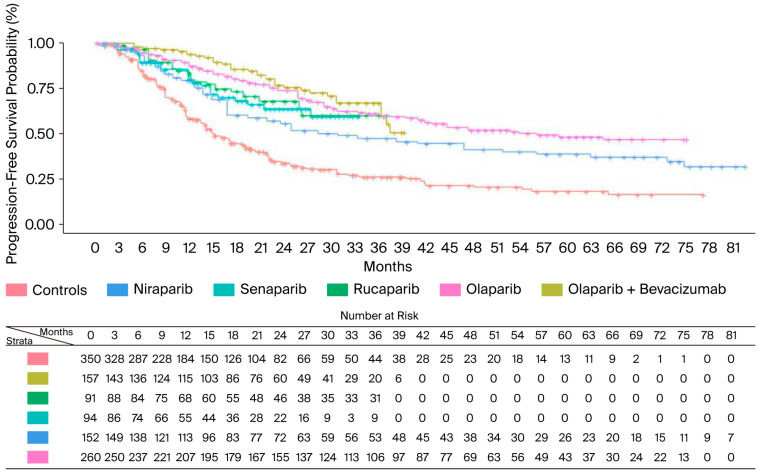
PFS of PARPi in BRCA+ population. After reconstruction of individual patient data (IPD) from five trials, the following PFS KM curves were generated: olaparib + bevacizumab (n = 157; in gold); rucaparib (n = 91; in green); senaparib (n = 94; in turquoise); niraparib (n = 152; in light blue); and olaparib (n = 260; in pink). The control arm (n = 350; in red) has been generated by pooling IPD from the control arms of the five trials. Abbreviations: n, number of patients.

In the BRCA+ population, maintenance therapy with PARPi significantly improved both HR and median PFS. Figure 2 shows cumulative KM curves for PFS in the BRCA+ population.

Indirect comparisons of the PFS benefits of different PARPi indicated that the combination of olaparib and bevacizumab was the most effective treatment for this patient subgroup (HR = 0.27; 95% CI: 0.19–0.39), with the median PFS not yet being reached at a median follow-up of 40 months and a median restricted mean survival time (RMST) of 28.6 months (95% CI: 27.19–30.00). Olaparib monotherapy was the second most effective treatment in terms of PFS benefit, followed by rucaparib, senaparib, and niraparib. Compared to the other PARPi, niraparib showed significantly worse survival outcomes, compared to the best in class, with a median PFS of 30.9 months (95% CI: 21.9–51.8) and a median RMST of 23.3 months (95% CI: 21.53–25.06), which was 5.3 months lower than the olaparib + bevacizumab RMST (HR for PFS 0.51; 95% CI: 0.33–0.79). As olaparib and niraparib are widely used in clinical practice, we have also investigated the difference between these two PARPinhibitors at longer follow-up times (RMST truncated at 75 months). Olaparib still remained the most effective PARPi, with an RMST of 48.94 months (95% CI: 45.26–52.62), which was 8.5 months longer than that of niraparib (HR = 0.65; 95% CI: 0.46–0.94).

**Figure 3:**
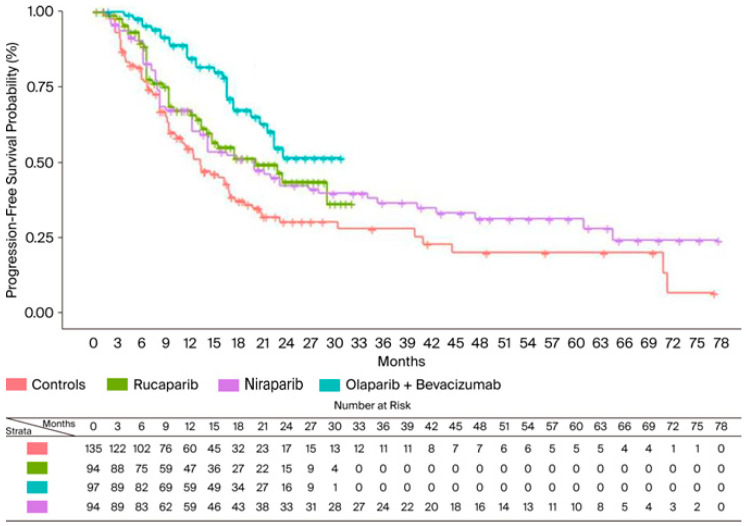
PFS of PARPi in BRCA−/HRD+ population. After reconstruction of individual patient data (IPD) from three trials, the following PFS KM curves were generated: rucaparib (n = 94; in green); olaparib + bevacizumab (n = 97; in turquoise); and niraparib (n = 94; in violet). The control arm (n = 135; in red) has been generated by pooling IPD from the control arms of the three trials. Abbreviations: n, number of patients.

In the BRCA−/HRD+ population, KM curves for PFS were available only from ATHENA, PAOLA1, and PRIMA studies. In this subgroup, the combination of olaparib + bevacizumab was superior to both rucaparib (HR = 0.58; 95% CI: 0.33–1.03) and niraparib (HR = 0.56; 95% CI: 0.33–0.97) in terms of PFS benefit. Olaparib + bevacizumab presented an RMST of 23 months (95% CI: 20.98–25.02), which was 3.7 months longer than rucaparib and 4.2 longer than niraparib. Cumulative KM curves for PFS in the BRCA−/HRD+ population are depicted in Figure 3.

**Figure 4:**
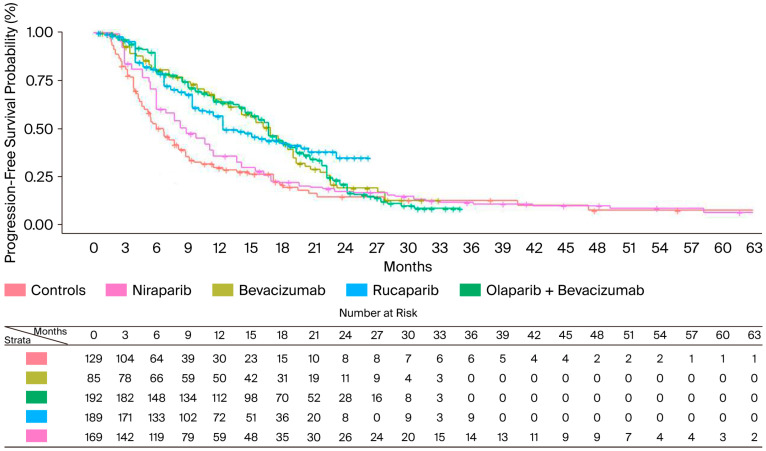
PFS of PARPi in BRCA−/HRD− population. After reconstruction of individual patient data (IPD) from three trials, the following PFS KM curves were generated: bevacizumab (n = 85; in gold); olaparib + bevacizumab (n = 192; in green); rucaparib (n = 189; in light blue); niraparib (n = 169; in violet). Control arm (n = 129; in red) has been generated by pooling IPD from the control arms of the three trials. Abbreviations: n, number of patients.

The BRCA−/HRD− subgroup experienced a lower survival benefit from PARPi compared to other subgroups. In this population, the median PFS ranged from 16.74 months with olaparib + bevacizumab (95% CI: 15.13–18.78) to 8.92 months with niraparib (95% CI: 7.78–11.15). Indirect comparisons between PARPi indicated that there were no significant differences among these regimens, but niraparib tended to perform worse than the others (HR = 1.57; 95% CI: 1.07–2.31 compared to rucaparib). In terms of RMST, olaparib + bevacizumab and rucaparib produced similar results, with an RMST of 15 months—4 months longer than that of niraparib. As the control arm of the PAOLA1 study (i.e., bevacizumab monotherapy) showed a significant difference in PFS compared to placebo controls in this population, we decided to include it in the indirect comparison rather than pooling these results with those from placebo-controlled arms of the other RCTs. Our results indicate that the addition of olaparib to bevacizumab did not improve survival compared to bevacizumab monotherapy (HR = 1.0; 95% CI: 0.67–1.51). PFS results in the BRCA−/HRD− population are shown in Figure 4.

In an exploratory analysis of OS, KM curves were available for SOLO1, PAOLA1, and PRIMA RCTs. Cumulative KM curves for OS in the BRCA+, BRCA−/HRD+, and BRCA−/HRD− populations are available as Supplementary Figure S2. In the BRCA+ population, the use of olaparib produced a significant OS advantage over placebo (HR = 0.58; 95% CI: 0.45–0.75), but the addition of bevacizumab did not produce any additional increase in OS (Supplementary Figure S2A). On the other hand, niraparib maintenance failed to demonstrate a survival benefit compared to placebo (HR = 1.04; 95% CI: 0.79–1.36). In the HRD+/BRCA− population, the survival advance was not as evident as in the BRCA+ subgroup. Olaparib + bevacizumab demonstrated a slightly better result in terms of OS compared to placebo (HR = 0.70; 95% CI: 0.48–1.0) and to niraparib (HR vs. placebo = 0.8 95%; CI: 0.56–1.15) (Supplementary Figure S2B). In the BRCA−/HRD− population, the survival benefit of PARPi (+/− bevacizumab) was not evident compared to placebo (Supplementary Figure S2C).

### 3.2. Adverse Drug Reactions Analysis

The analysis of any-grade ADR showed that most PARPi were well tolerated, with no substantial difference in the incidence of any-grade ADR compared to placebo. Incidences of any-grade and severe ADR (higher than grade 3, >G3) are shown in Supplementary Figures S3 and S4, respectively.

**Figure 5:**
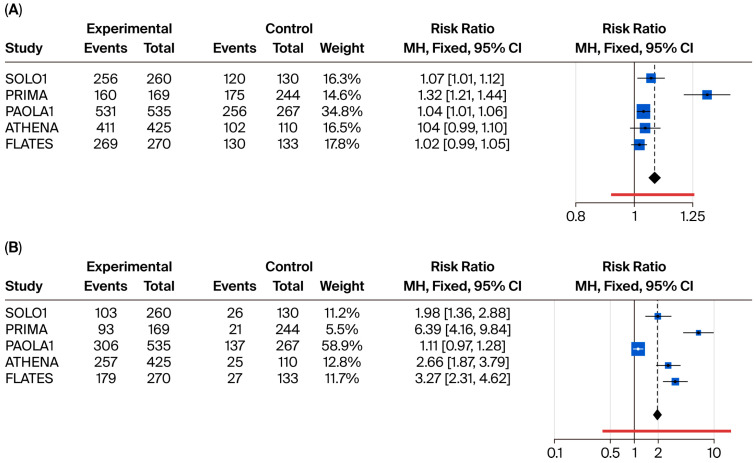
Binary meta-analysis comparing incidence of any-grade ADR (panel (**A**)) and severe > G3 ADR (panel (**B**)) in patients treated with olaparib (SOLO1 study), niraparib (PRIMA study), olaparib + bevacizumab (PAOLA1 study), rucaparib (ATHENA study), and senaparib (FLAMES study) compared to placebo. Risk ratio estimates with 95% CI and crude rates in the active and control arms are reported.

In the meta-analysis of safety profiles, niraparib showed a worse safety profile with an RR of developing any-grade ADR equal to 1.32 (95% CI: 1.21–1.44) compared to placebo. When severe ADR has been considered in the analysis, olaparib + bevacizumab presented the best safety profile with a RR of >G3 ADR equal to 1.11 (95% CI: 0.97–1.28) compared to placebo and significantly lower RR compared to other treatments. On the contrary, niraparib also showed the worst safety profile in terms of severe ADR, with an RR of 6.39 (95% CI: 4.16–9.84) compared to placebo and a significantly higher RR for severe ADR compared to all other regimens. Results are reported in Figure 5.

**Figure 6:**
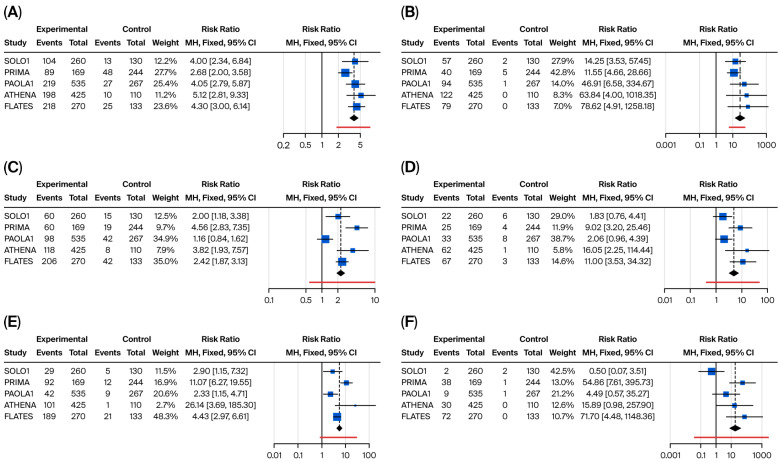
Binary meta-analysis comparing incidence of hematological ADR in patients treated with olaparib (SOLO1 study), niraparib (PRIMA study), olaparib + bevacizumab (PAOLA1 study), rucaparib (ATHENA study), and senaparib (FLAMES study) compared to placebo. Risk ratio estimates with 95% CI and crude rates in the active and control arms are reported for anemia (**A**,**B**), neutropenia (**C**,**D**), and thrombocytopenia (**E**,**F**), respectively, for both any-grade (left part of the panel) and severe occurrence of these ADR (≥G3, right part of the panel).

**Figure 7:**
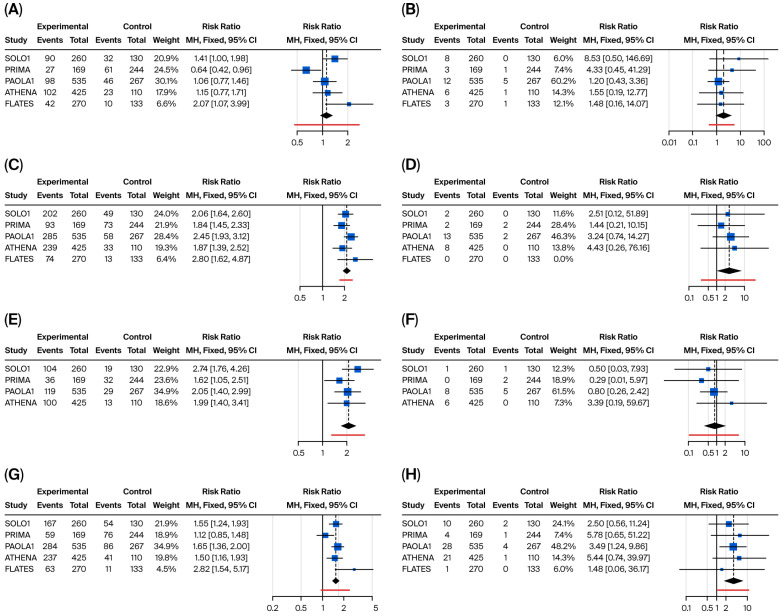
Binary meta-analysis comparing incidence of gastrointestinal ADR in patients treated with olaparib (SOLO1 study), niraparib (PRIMA study), olaparib + bevacizumab (PAOLA1 study), rucaparib (ATHENA study), and senaparib (FLAMES study) compared to placebo. Risk ratio estimates with 95% CI and crude rates in the active and control arms are reported for diarrhea (**A**,**B**), nausea (**C**,**D**), vomiting (**E**,**F**), and fatigue or asthenia (**G**,**H**), respectively, for any-grade (left part of the panel) and severe occurrence of these ADR (≥G3, right part of the panel).

Network meta-analysis revealed significant differences in specific ADR patterns. The results for the main hematological and gastrointestinal/systemic ADRs are presented in Figure 6 and Figure 7, respectively.

Hematological toxicities (particularly anemia and thrombocytopenia) were the predominant major concerns linked to the use of PARPi, particularly anemia, which is a class effect with homogeneous incidence alongside the different PARPi across all regimens. Olaparib demonstrated the most favorable profile in terms of hematological toxicity, with significantly reduced incidence of any-grade neutropenia compared to senaparib, rucaparib and niraparib (RR = 0.44 (95% CI: 0.22–0.89)), and a significantly reduced incidence of thrombocytopenia compared to niraparib and rucaparib (RR = 0.11 (95% CI: 0.01; 0.97)). Overall, senaparib was associated with the highest incidence of severe anemia (grade ≥ 3: 29%), thrombocytopenia (grade ≥ 3: 27%), and neutropenia (grade ≥ 3: 25%), reflecting the myelosuppressive effects of the treatment. Despite dose optimization strategies, niraparib still demonstrated significant hematological toxicity, with grade ≥ 3 anemia occurring in 24% of patients, and significantly increased incidence of thrombocytopenia and neutropenia compared to olaparib (RR for severe thrombocytopenia olaparib vs. niraparib = 0.01 (95% CI: 0.00–0.15); RR for severe neutropenia olaparib vs. niraparib 0.20 (95% CI: 0.05–0.79)).

Gastrointestinal and systemic toxicities, although with a lower incidence than hematological toxicities, were still an important major category of concern. Niraparib demonstrated the best safety profile in terms of nausea (RR compared to senaparib = 0.66; 95% CI: 0.36–1.20), diarrhea (RR compared to senaparib = 0.31; 95% CI: 0.14–0.67), and vomiting (RR compared to olaparib = 0.59; 95% CI: 0.32–1.10), while senaparib and olaparib exhibited the poorest profiles. One of the major ADRs that has a significant impact on the quality of life of ovarian cancer patients is asthenia and fatigue. Niraparib demonstrated a lower incidence of fatigue, which was significantly lower than that observed with senaparib (RR = 0.40; 95% CI: 0.20–0.77) and olaparib + bevacizumab (RR = 0.68; 95% CI: 0.49–0.95). However, these differences were less evident when considering severe fatigue and asthenia.

## 4. Discussion

In this study, we conducted an indirect comparison of RCTs evaluating the efficacy and safety of PARPi, either as monotherapy or in combination with bevacizumab, across molecularly defined subgroups of ovarian cancer patients.

Consistent with prior evidence, our findings reinforce that the greatest benefit from PARPi maintenance is observed in patients with BRCA+ tumors [30]. In this subgroup, olaparib—either alone or in combination with bevacizumab—provided the most pronounced PFS benefit, with RMST analyses demonstrating durable efficacy beyond 6 years of follow-up. Notably, niraparib seems to underperform compared to other agents, both in early and long-term analyses. Nonetheless, the approach is limited by its dependence on the availability of subgroup-specific survival curves, which are not consistently reported across trials. In our case, the partial availability of KM curves also hinders the possibility of investigating PFS in patients with different risk classes. In the PRIMA study, for example, R0 patients were not enrolled; therefore, the population was entirely high risk when compared to other PARPi, which could introduce a selection bias and could have a negative impact on the results of the indirect comparison of niraparib with other PARPi. Risk class-based analysis could help to better characterize the efficacy of PARPi and should be further explored in future studies. Nevertheless, our heterogeneity analysis, based on the PFS of the control arms in different studies, indicated that although all patients in the PRIMA study were high risk, they behaved similarly to patients enrolled in other RCTs when observed for PFS in a placebo-receiving setting.

Multiple phase III trials are ongoing, evaluating combinations of PARPi with immune checkpoint inhibitors (ICIs), anti-angiogenic agents, or chemotherapy, with key results expected between 2025 and 2030. Early-phase studies already suggest that integrating PARPi with ICIs or anti-angiogenesis agents yields promising antitumor activity, particularly in BRCA−mutated or HRD−positive disease [31].

In the BRCA−/HRD+ subgroup, the addition of bevacizumab to olaparib also demonstrated superior PFS outcomes compared to niraparib and rucaparib, with RMST gains of up to 4 months. These results highlight a possible synergistic interaction between PARPi and anti-angiogenic therapy in HRD−positive, BRCA wild-type tumors, a biologically plausible finding given the preclinical evidence of VEGF-mediated modulation of DNA repair pathways. In addition, in HRD patients, emerging evidence highlights the potential of novel maintenance strategies in ovarian cancer beyond conventional PARP inhibition. Preclinical studies demonstrate that PI3K inhibition enhances tumor sensitivity to PARPi by disrupting homologous recombination repair, underscoring the rationale for combination therapy; however, recent results from the EPIK-O/ENGOT-ov61 trial, combining alpelisib + olaparib, failed to demonstrate a significant PFS advantage in BRCA− patients [32].

Conversely, in the BRCA−/HRD− subgroup, PARPi efficacy was markedly reduced. PFS improvements were modest and did not translate into significant OS advantages. Importantly, the addition of olaparib to bevacizumab did not improve outcomes compared to bevacizumab alone, raising concerns about overtreatment in this molecularly defined population. The ongoing MITO 25 trial (NCT03462212) might provide additional direct evidence to this observation. This study compares the efficacy of carboplatin–paclitaxel and rucaparib maintenance to carboplatin–paclitaxel-bevacizumab and bevacizumab plus rucaparib maintenance also in HR proficient patients. Nevertheless, this population is orphaned by effective treatment strategies and is therefore the one in which biomarker-driven approaches are gaining traction. Lipid metabolism is emerging as a therapeutic target, with inhibitors of fatty acid synthase, cholesterol synthesis, and fatty acid oxidation showing potential to overcome resistance. Additionally, novel immunotherapy combinations—such as ICIs with bevacizumab, chemotherapy, PARPi, or antibody–drug conjugates like mirvetuximab soravtansine—have shown encouraging response rates in recurrent disease and may be beneficial also moved upfront in the patient’s therapeutic program. Finally, precision oncology is expanding with agents targeting specific genomic alterations, including larotrectinib (NTRK fusions) and selpercatinib (RET fusions), emphasizing the importance of biomarker testing in guiding individualized treatment [33].

While current clinical research predominantly aims to extend PFS, future studies should place stronger emphasis on OS and patient quality of life. Our exploratory OS analyses confirmed the long-term survival benefit of olaparib in BRCA+ patients, while the addition of bevacizumab did not confer further OS advantage despite its PFS benefit. In HRD+ patients without BRCA mutations, a modest OS improvement was observed with olaparib + bevacizumab, but this did not reach clear statistical significance. In the HRD− subgroup, no OS advantage was apparent for any PARPi regimen, underscoring the need for biomarker-driven treatment selection. Although the exploratory analysis of survival data provided some insights, the results and comparisons are limited by the data availability across individual trials and should be further explored in future studies.

Nowadays, the median OS in ovarian cancer chemo-radio treated is higher close to 50 months [34], but the vast majority of patients experience disease relapse within 3 years of diagnosis [3]. Thus, a clever long-term strategy is fundamental to taking advantage of the beneficial effect of each drug. Our data strongly support the essential role of pretreatment assessment of mutational status in order to offer a more comprehensive and personalized approach to each patient [35].

Women diagnosed with BRCA+/HRD− disease experience higher sensitivity to platinum-based chemotherapy and to PARPi and the longer median OS [36]. This subset of ovarian cancer patients should be considered for a PARP inhibitor as a first-line maintenance strategy, according to patient characteristics (i.e., comorbidities and concomitant medications) and drug safety profiles. The best setting for the use of bevacizumab will probably be the second line.

On the other hand, the majority of HRD+ patients without clinical contraindication should receive olaparib + bevacizumab due to the favorable safety profile and the strong efficacy data concerning this combination in this population.

The real unmet clinical need remains the HRD− patients, who experience lower drug sensitivity and the worst prognosis. According to a recent consensus, patients with high-risk disease should receive bevacizumab upfront, regardless of BRCA/HRD status [35]. Matching this statement and our data, the preferable strategy in HRD− patients may be the use of bevacizumab in first-line maintenance and, eventually, PARPi (i.e., rucaparib or niraparib, which are indicated in the all-comers population) as second-line maintenance.

Our comparative safety analysis revealed important differences among PARPi regimens. Niraparib was consistently associated with the highest rates of both all-grade and severe ADR, particularly hematological toxicities such as thrombocytopenia and neutropenia, despite dose optimization strategies. Senaparib also demonstrated substantial myelosuppressive activity, whereas olaparib displayed the most favorable hematological safety profile. When combined with bevacizumab, olaparib maintained a relatively low risk of severe ADRs, highlighting its favorable risk–benefit balance in appropriate patient subgroups. Non-hematological toxicities were generally less frequent, but gastrointestinal events and fatigue remained clinically relevant. Interestingly, niraparib was associated with fewer gastrointestinal toxicities and a lower incidence of fatigue compared to other PARPi, which could partially mitigate its less favorable hematological profile, and it may constitute a significant advantage in terms of quality of life in patients with active lifestyle or have returned to work.

In our analysis we used the IPDfromKM methodology to reconstruct IPD from published KM survival curves. This method is increasingly applied in oncology to facilitate indirect survival comparisons. In this study, it allowed us to pool patients derived from control arms of RCTs, thereby increasing the size and robustness of the common comparator arm. In a separate analysis [37], we verified the excellent quality of the reconstruction performed by the IPDfromKM method on these datasets. Important advantages of this technique include the preservation of event timing, a feature usually lost in conventional meta-analyses that rely on binary endpoints, and the possibility of generating overlaid KM plots that improve visualization and interpretability of outcomes. Despite efforts to harmonize control arms and reconstruct IPD, residual heterogeneity across trials cannot be excluded, particularly regarding patient selection criteria, prior chemotherapy response, and follow-up duration. Moreover, mature OS data are lacking for several agents, and ongoing follow-up will be needed to validate long-term survival effects. Furthermore, the subgroup analysis is also constrained by the availability of data and the robustness of those data. Finally, real-world data are essential to complement RCT evidence, especially given differences in toxicity management outside clinical trial settings.

## 5. Conclusions

In conclusion, despite the methodological limitations of indirect comparisons, this comparative analysis confirms that the extent of the benefit from PARP inhibition is strongly dependent on BRCA and HRD status. Olaparib-based regimens provide the greatest efficacy, with a favorable safety profile in BRCA+ and HRD+ populations, whereas the clinical utility of PARPi in HRD-negative patients remains questionable. The differences observed in our studies are partially consistent with the ESMO clinical guidelines for the treatment of ovarian cancer. Our study confirms the ESMO recommendation for the use of olaparib-based regimens in patients with BRCA−positive tumors. However, further evidence from head-to-head RCTs is needed to support the introduction of PARPi maintenance in patients with HRD-negative/BRCA-negative tumors [38]. Future work should focus on optimizing patient selection, including analysis based on class of risk, identifying predictive biomarkers beyond BRCA/HRD status, and integrating PARPi with emerging therapeutic strategies.

## Data Availability

The data presented in this study are available in the article and in the Supplementary Material.

## Supplementary Materials

The following supporting information can be downloaded at https://www.mdpi.com/article/10.3390/cancers17223714/s1, Figure S1: Visual representation of indirect treatment comparisons of PFS in BRCA+, BRCA−/HRD+, and BRCA−/HRD− cohorts of the included trials; Figure S2: OS of PARPi after reconstruction of individual patient data (IPD) from three trials available: SOLO1, PAOLA1, and PRIMA RCTs in BRCA+, BRCA−/HRD+, and BRCA−/HRD− cohorts; Figure S3: Graphical representation of incidence of any-grade ADR in SOLO1, PRIMA, PAOLA1, ATHENA, and FLAMES studies; Figure S4: Graphical representation of incidence of severe ADR ≥ G3 in SOLO1, PRIMA, PAOLA1, ATHENA, and FLAMES studies.
