# Supplementary material for: Comparative Analysis of Maintenance Treatments in Patients with Newly Diagnosed Advanced Ovarian Cancer After First-Line Platinum-Based Regimens
Source: Cancers (Basel). 2025 Nov 20;17(22):3714. doi: 10.3390/cancers17223714 (PMC12651439; doi:10.3390/cancers17223714)
Supplement: Supplementary file 1 [file cancers-17-03714-s001.zip › cancers-3921548-supplementary.pdf]

## Supplementary material

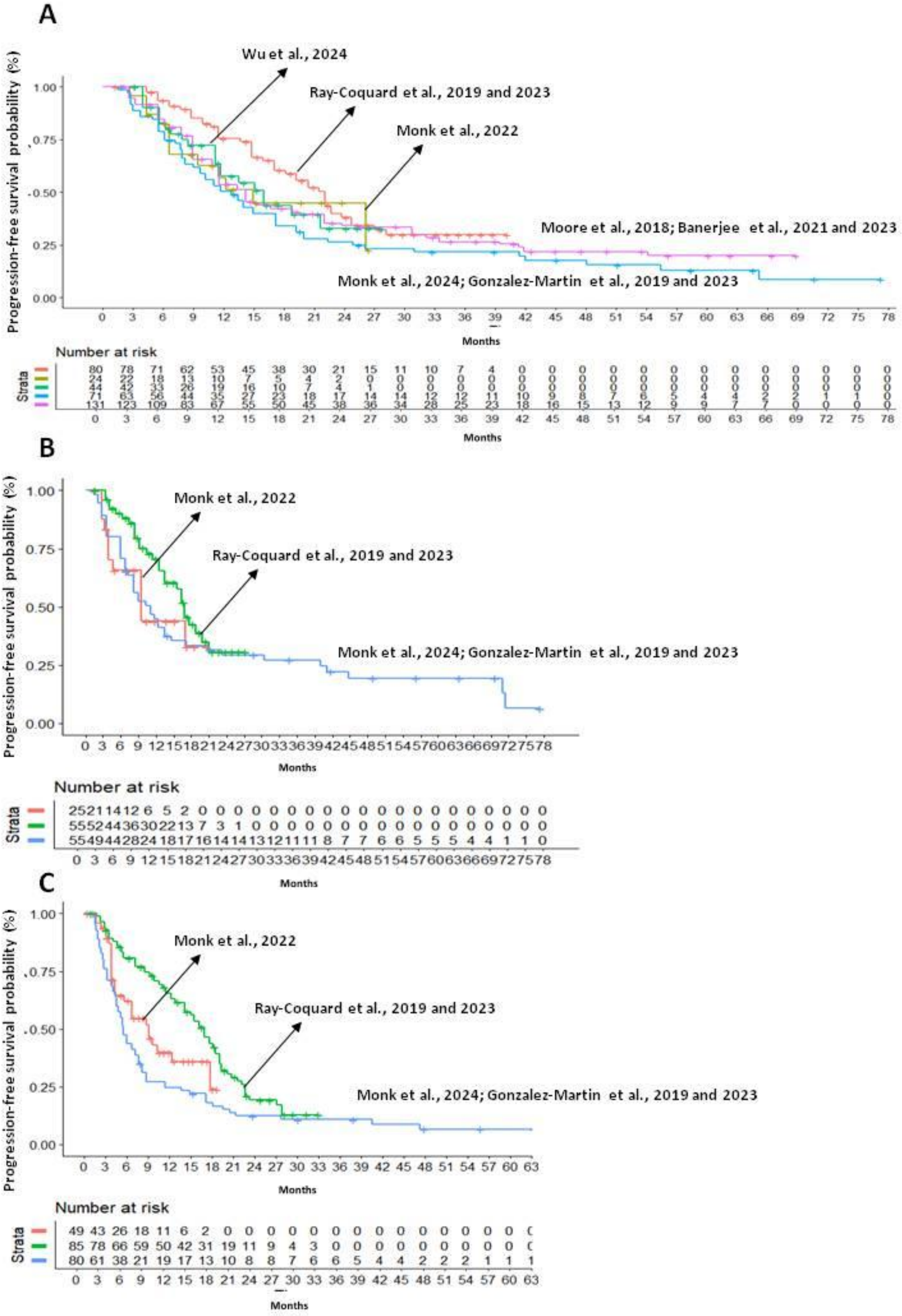

Supplementary Figure S1. Panel A: Kaplan–Meier curves of PFS generated after reconstructing patient-level data from the placebo-treated control arms in BRCA+ cohort of the included trials. PAOLA1 (n = 80; in red), ATHENA (n = 24; in gold), FLAMES (n = 44; in green), PRIMA (n = 71; in turquoise), and SOLO1 (n = 131; in pink). Panel B: Kaplan–Meier curves of PFS generated after reconstructing patient-level data from the placebo-treated control arms in BRCA-/HRD+ cohort of the included trials. ATHENA (n = 25; in red), PAOLA1 (n = 55; in green), and PRIMA (n = 55; in turquoise). Panel C: Kaplan–Meier curves of PFS generated after reconstructing patient-level data from the placebo-treated control arms in BRCA-/HRD- cohort of the included trials. ATHENA (n = 49; in red), PAOLA1 (n = 85; in green), and PRIMA (n = 80; in turquoise). Endpoint: progression-free survival (PFS), time in months. Abbreviations: n, number of patients.

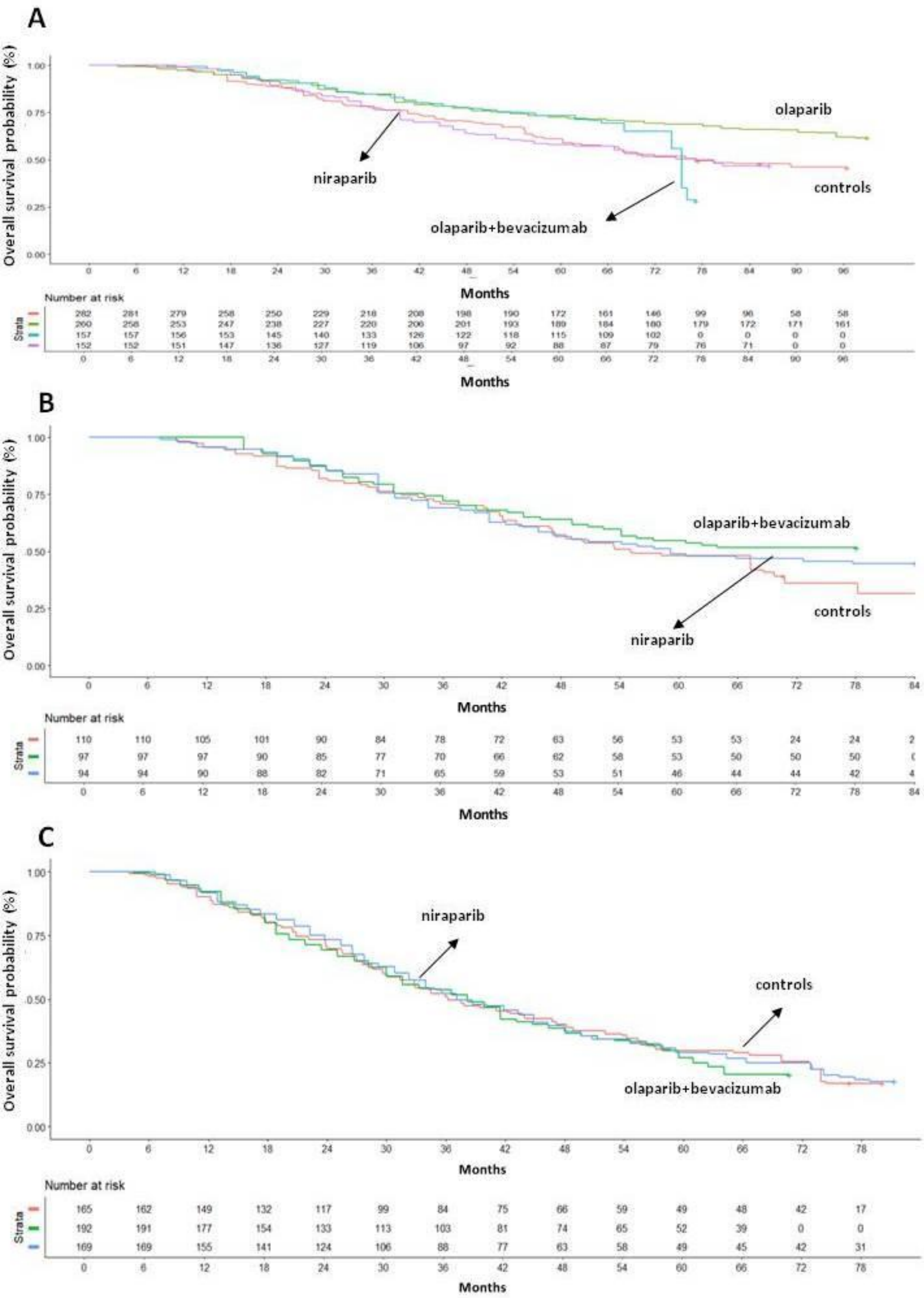
A
Overall survival probability (%)
olaparib
niraparib
controls
olaparib+bevacizumab
Months
Number at risk
Strata
B
Overall survival probability (%)
olaparib+bevacizumab
controls
niraparib
Months
Number at risk
Strata
C
Overall survival probability (%)
niraparib
controls
olaparib+bevacizumab
Months
Number at risk
Strata

*Supplementary Figure S2. OS of PARP inhibitors after reconstruction of individual patient data (IPD) from three trials available: SOLO1, PAOLA 1 and PRIMA RCTs. Panel A: Kaplan–Meier curves of OS generated after reconstructing patient-level data in BRCA+ cohort of the included trials. Olaparib (n = 260; in gold), olaparib + bevacizumab (n = 157; in light blue) and niraparib (n = 152; in pink). Control arm (n=282; in red) has been generated pooling IPD from the control arms of the three trials. Panel B: Kaplan–Meier curves of OS generated after reconstructing patient-level data in BRCA-/HRD+ cohort of the included trials. olaparib + bevacizumab (n = 97; in green) and niraparib (n = 94; in turquoise). Control arm (n=110; in red) has been generated pooling IPD from the control arms of the two trials. Panel C: Kaplan–Meier curves of OS generated after reconstructing patient-level data in BCRA-/HRD- cohort of the included trials. olaparib + bevacizumab (n = 192; in green) and niraparib (n = 169; in turquoise). Control arm (n=165; in red) has been generated pooling IPD from the control arms of the two trials. Endpoint: overall survival (OS), time in months. Abbreviations: n, number of patients.*

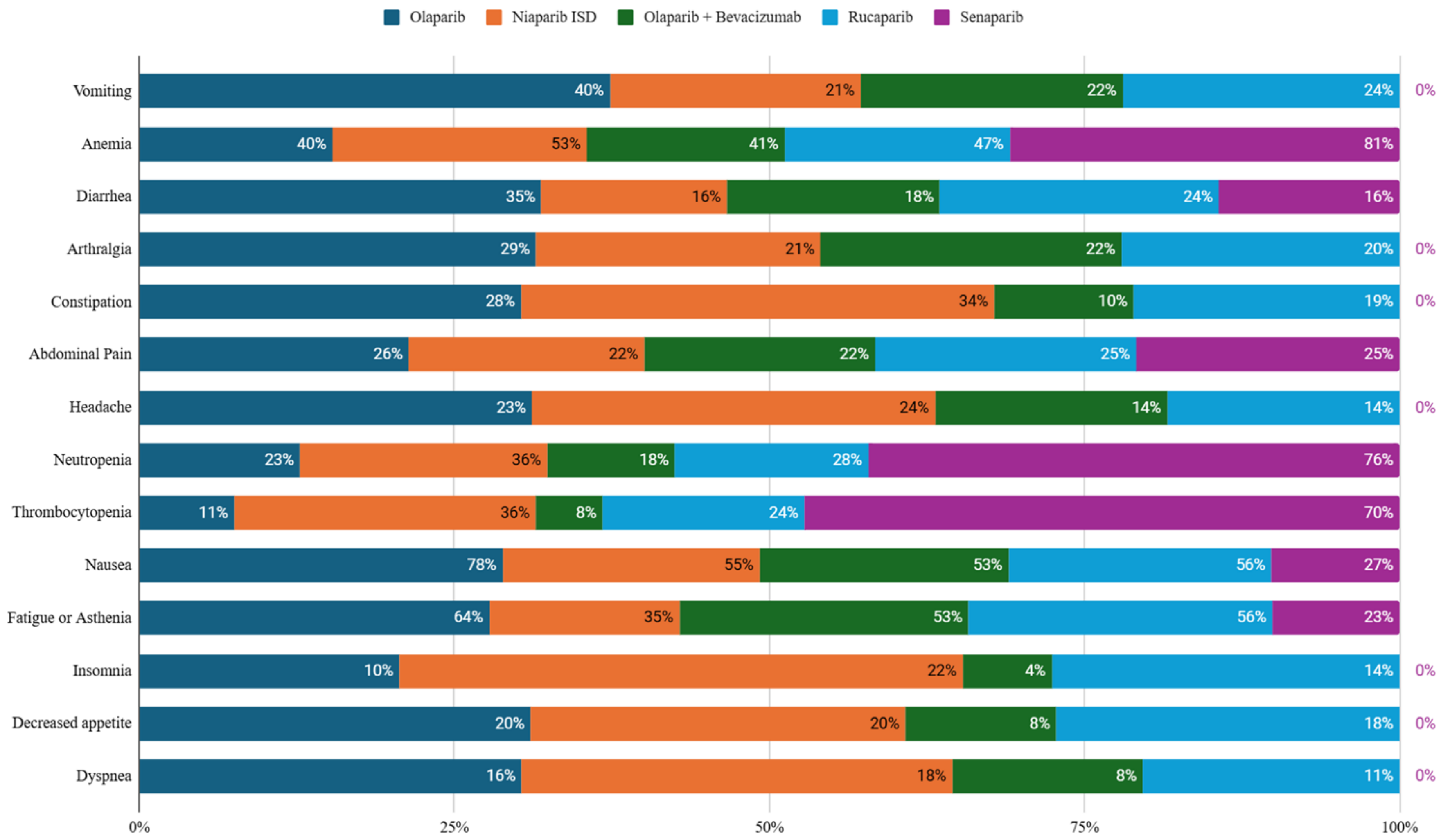


*Supplementary Figure S3. Incidence of any grade in SOLO1, PRIMA, PAOLA1, ATHENA and FLAMES studies are reported in blue, orange, green, light blue and purple bar, respectively.*

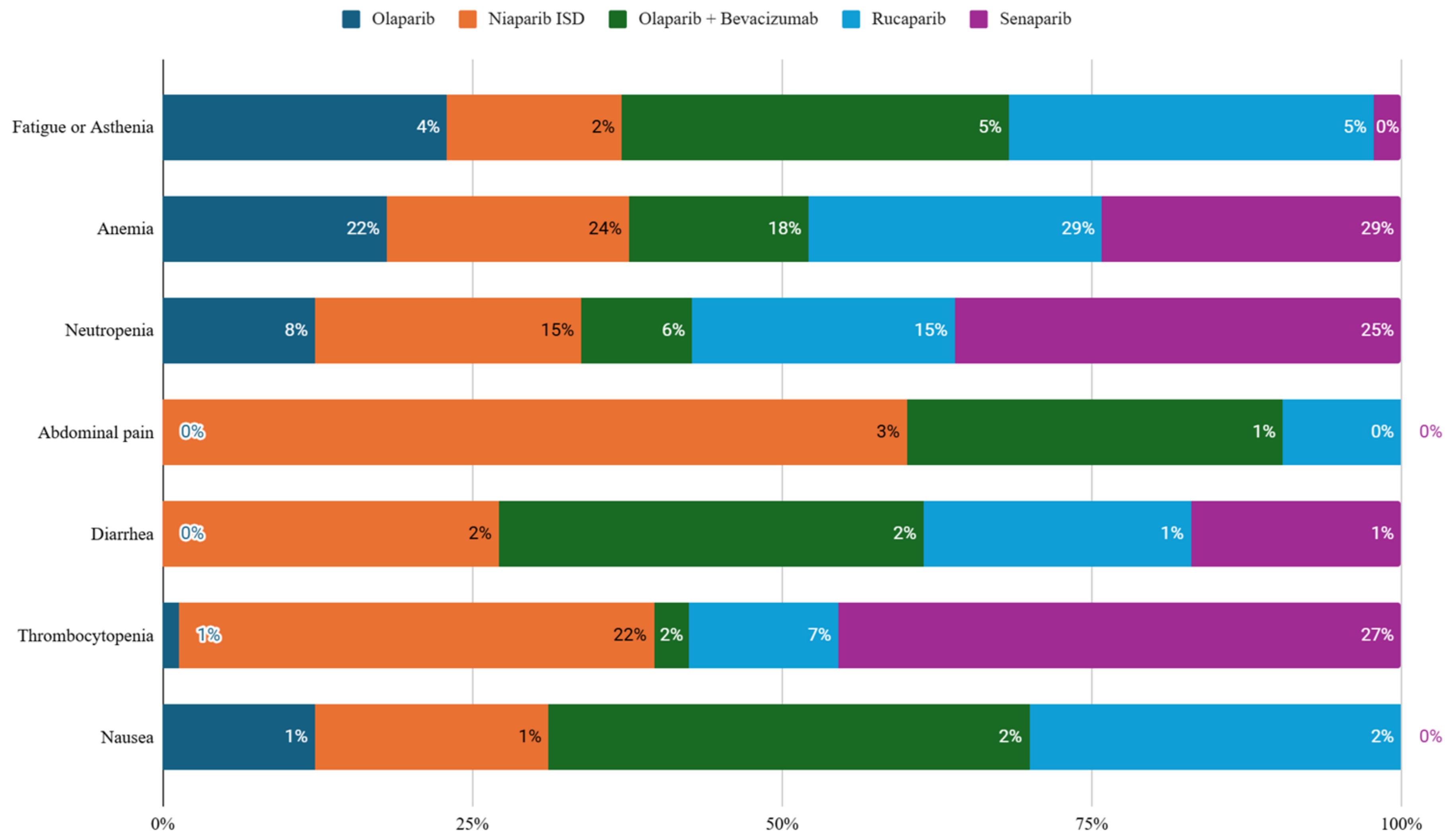


*Supplementary Figure S4. Incidence of severe ADR ≥G3 in SOLO1, PRIMA, PAOLA1, ATHENA and FLAMES studies are reported in blue, orange, green, light blue and purple bar, respectively.*
